# Molecular simulation of gases competitive adsorption in lignite and analysis of original CO desorption

**DOI:** 10.1038/s41598-021-91197-0

**Published:** 2021-06-03

**Authors:** Jing Zhang, Jiren Wang, Chunhua Zhang, Zongxiang Li, Jinchao Zhu, Bing Lu

**Affiliations:** 1 College of Safety Science & Engineering, Liaoning Technical University, Fuxin, 123000 Liaoning China; 2 Key Laboratory of Mine Thermodynamic Disaster & Control of Ministry of Education, Huludao, 125105 Liaoning China

**Keywords:** Atomic and molecular physics, Coal, Chemical physics

## Abstract

To study the adsorption characteristics of CO, CO_2_, N_2_, O_2_, and their binary-components in lignite coal, reveal the influence of CO_2_ or N_2_ injection and air leakage on the desorption of CO in goafs, a lignite model (C_206_H_206_N_2_O_44_) was established, and the supercell structure was optimized under temperatures of 288.15–318.15 K for molecular simulation. Based on molecular dynamics, the Grand Canonical Monte Carlo method was used to simulate the adsorption characteristics and the Langmuir equation was used to fit the adsorption isotherms of gases. The results show that for single-components, the order of adsorption capacity is CO_2_ > CO > O_2_ > N_2_. For binary-components, the competitive adsorption capacities of CO_2_ and CO are approximate. In the low-pressure zone, the competitive adsorption capacity of CO_2_ is stronger than that of CO, and the CO is stronger than N_2_ or O_2_. From the simulation, it can be seen that CO_2_, N_2_ or O_2_ will occupy adsorption sites, causing CO desorption. Therefore, to prevent the desorption of the original CO in the goaf, it is not suitable to use CO_2_ or N_2_ injection for fire prevention, and the air leakage at the working faces need to be controlled.

## Introduction

CO is a toxic and harmful gas in a coal mine, which affects the health of miners and restricts the production of coal mines^1^. For the generation of CO, it is generally considered that CO comes from the oxidation and spontaneous combustion of coal or mine explosion^2,3^, and no attention has been paid to the desorption of original CO in coal. Therefore, CO is widely used as an index gas to judge spontaneous combustion in many coal mines of China^4^. However, in recent years, it has been proved that spontaneous combustion of coal is not the only source of CO. In many low-rank coal mines, CO continues to exceed the standard and no signs of spontaneous combustion have been found. The literature shows the CO comes from the coal formation stage in these mines, instead of oxidation or spontaneous combustion^5^. Affected by mining, part of residual broken coal is left in the goaf, and the CO can desorb under various conditions and spread to the working face, which brings great difficulties to coal mine work. Coal is a porous material with surface area and pore volume, which provides conditions for gas adsorption. Therefore, understanding the CO adsorption mechanism and the adsorption competitive relationship between CO, O_2_, N_2_, and CO_2_ is of great significance to the control of CO in working faces.

Gases present in coal mainly enter through physical adsorption. The adsorption capacity of gas in coal is mainly affected by coal rank^6^, pore structure^7,8^, pressure and temperature^9^, moisture content^10^, functional groups^11^. Many scholars have made studies on coal adsorption gases. Tang et al.^12^ and Meng et al.^13^ studied the adsorption characteristics of coal to supercritical CO_2_ and verified the adaptability of the Langmuir equation to the fitting of coal adsorption isotherms, and the adsorption form of supercritical CO_2_ was analyzed. Zhang et al.^14^ and Wu et al.^15^ used a Monte Carlo-based molecular simulation method to study the effect of moisture content on coal adsorption of methane. Zhou et al.^16^ used molecular simulation methods to study the competitive adsorption characteristics of CO_2_/CH_4_/H_2_O on lignite, while Yu et al.^17^ further studied the competitive adsorption characteristics of CO_2_/CH_4_/H_2_O in different coal ranks. Gao et al.^18^ studied the adsorption characteristics of CO_2_/CH_4_/N_2_/H_2_O single-components and multi-components on lignite. For the adsorption of CO on coal, related research is relatively scarce. Zhu et al.^19^ conducted experiments to study the generation and release laws of the original CO in coal seams and verified the existence of other CO sources besides spontaneous combustion. Zhang et al.^20^ also analyzed the CO formation and release rules in mines without spontaneous combustion, and conducted a targeted study on the influence of inertinite and vitrinite on CO adsorption in different coal ranks. Sharma et al.^21^ investigated the shale gas adsorption and diffusion in inorganic nanopores by molecular simulation. Lin et al.^22–25^ studied methane displacement and shale gas molecular dynamics simulation and obtained adsorption characteristics of the gas on graphite and carbon nanotubes, the law of entropy change and enthalpy change, and free energy change in the process of adsorption or displacement. Wang et al.^26^ discussed the direct air capture of low concentration CO_2_ by 5A zeolite adsorbent bed combined GCMC and FVM method. Li et al.^27,28^ discussed the superior selective CO_2_ adsorption of C_3_N pores by GCMC and DTF simulations.

The research of scholars mainly focused on the competitive adsorption of CH_4_, N_2_, and CO_2_ by experiments or molecular simulation methods. A few scholars have studied the adsorption characteristics of CO in coal through experiments but did not use molecular simulation to further study the mechanism and the adsorption competitive relationship of CO with other gases on lignite molecules. Here, by using molecular simulation technology based on molecular mechanics and dynamics, it is found that in the mining process, the leakage of fresh air and the injection of fire-prevention gases in the goaf are the reasons for the desorption and diffusion of the original CO. To more clearly compare the adsorption competitive characteristics and capacities of CO and other gases in lignite, this paper establishes supercell structures of lignite and adds CO to the adsorption simulation system for the first time, simulating CO, CO_2_, N_2_, O_2_ and their binary-components. The disadvantages of traditional fire-prevention work for the mines with the original CO desorption are analyzed. The results of this research have played an important guiding role in CO prevention and control in coal mines.

## Simulation condition

### Establishment of the coal model

**Figure 1:**
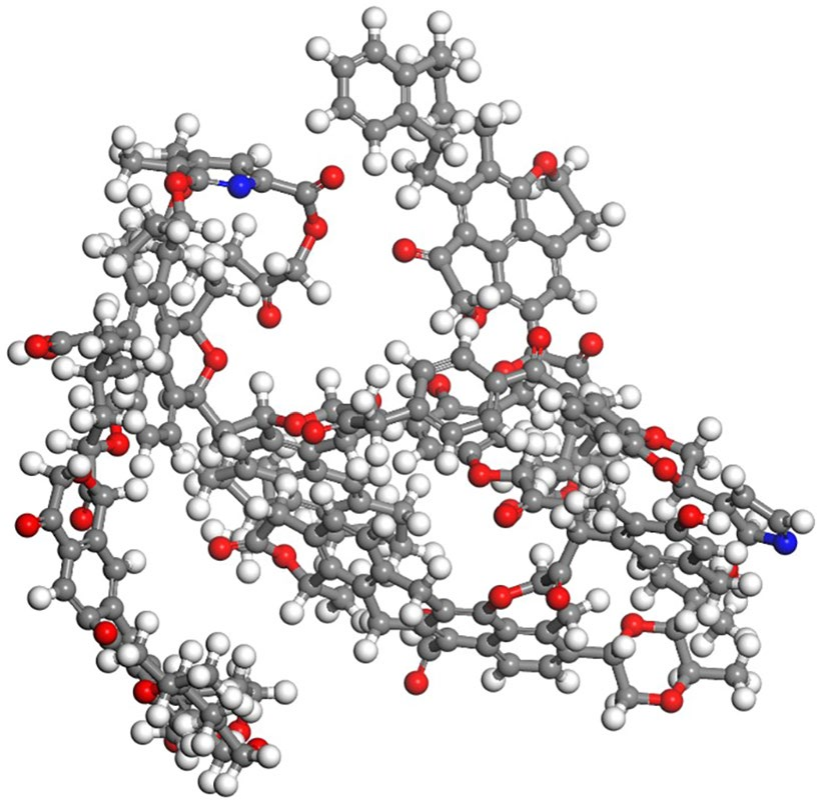
The basic unit of lignite molecular C_206_H_206_N_2_O_44_ (C: grey; O: red; N: blue; H: white).

**Figure 2:**
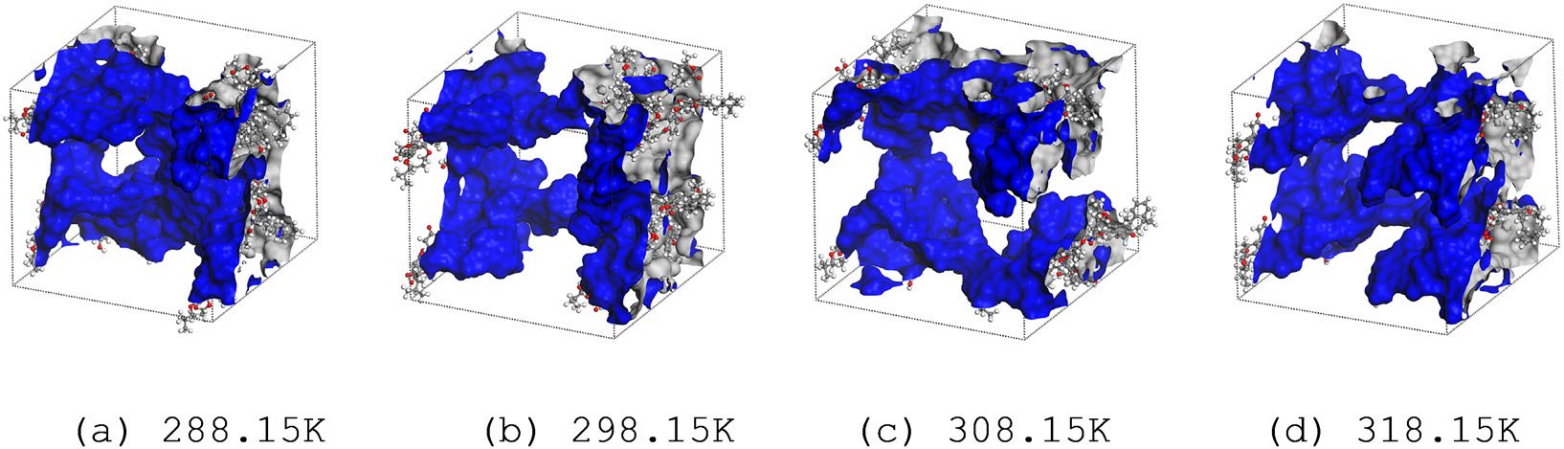
Supercell structures and Connolly surfaces of lignite at different temperatures (Blue areas are Connolly surfaces).

The basic unit model of lignite coal (C_206_H_206_N_2_O_44_)^29^ is established as shown in Fig. 1. The literature shows that the structure of coal is closely related to temperature, so the normal working temperatures (288.15, 298.15, 303.15, and 313.15 K) of the mine is selected, and the 3D Triclinic Lattice type with fixing angle (α: 90°, β: 90°, γ: 90°) is used. By using the "Forcite" module, the coal unit cell is optimized and annealed, and the charge balance method (QEq) is used to calculate the charge distribution. The unit cell size is a = 41.80 Å, b = 39.46 Å, c = 37.71 Å. Considering that the adsorption of gas molecules may lead to a pore-blocking phenomenon with pore volume decreases in reality^30^, a probe with a radius of 1.6 Å was used to determine the differences of pore structures. In the simulation, whether the adsorbed molecules cause a pore-blocking phenomenon and reduce the adsorption capacity is controlled by the simulation soft. The Connolly free volume of the optimal supercells at four temperatures are 46,245.14 Å^3^, 46,321.85 Å^3^, 46,206.47 Å^3^, and 46,266.11 Å^3^, respectively, and the surface areas are 7633.82 Å^2^, 7603.38 Å^2^, 7663.65 Å^2^, and 7195.66 Å^2^, respectively. As shown in Fig. 2, it can be seen that temperature has a significant influence on the structure of coal molecular unit cells^31^. Therefore, it is necessary to optimize annealing at different temperatures. When the temperature reaches 308.15 K, the supercell structure has the smallest Connolly free volume and the largest surface area.

Here, the adsorption of CO, CO_2_, O_2_, N_2_, and their binary-components were simulated on the lignite. The adsorption characteristics were determined. By comparing the adsorption characteristics and capacity of CO and other gases, the desorption mechanism of CO and the influences of other gases were analyzed in mines without spontaneous combustion danger.

### Relationship between fugacity and pressure

#### Fugacity of single-components

**Figure 3:**
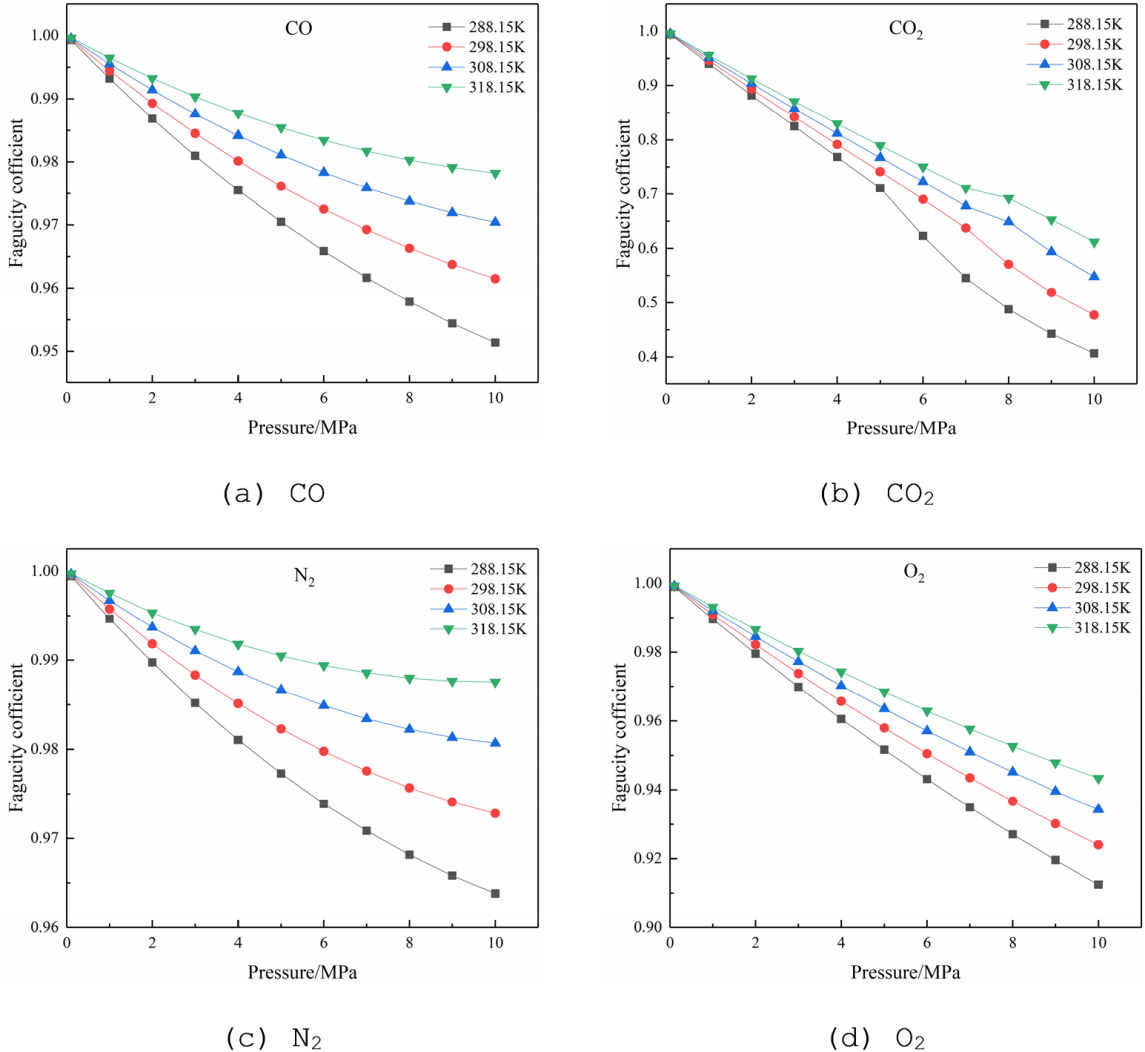
The relationship between the fugacity coefficient and pressure of single-components at different temperatures.

The temperatures are specified as 288.15, 298.15, 308.15, and 318.15 K, respectively, and the pressures are between 0 and 10 MPa. The conversion between pressure and fugacity is realized by using the Peng-Robinson formula. The physical properties of the four gases are shown in Table 1. The relationship between the fugacity of each gas and the pressure is shown in Fig. 3, where the fugacity coefficient is the ratio of fugacity to pressure.

**Table 1:** Physical properties of the gases.

Physical properties	CO	CO_2_	N_2_	O_2_
Molar mass *M*/(g mol^−1^)	28.01	44.01	28.01	32.00
Critical temperature *T*_*c*_/K	132.86	304.12	126.19	154.58
Critical pressure *P*_*c*_/MPa	3.49	7.38	3.40	5.04
Acentric factor	0.05	0.22	0.04	0.022

The fugacity of a single-component is mainly affected by temperature and pressure. Under the same temperature condition, the fugacity coefficient decreases with the increase of pressure; under the same pressure condition, the fugacity coefficient increases with the increase of temperature. In the pressures of 0–10 MPa and the temperatures of 288.15–318.15 K, the fugacity coefficients of CO, O_2_, and N_2_ have relatively small changes, between 0.9 and 1.0; while the fugacity coefficient of CO_2_ changes greatly, between 0.4 and 1.0. This change is mainly due to the high critical temperature of CO_2_, which is 304.13 K. Therefore, at 288.15 and 298.15 K, when the pressure exceeds critical pressure 7.38 MPa, CO_2_ changes from a gas to a liquid, the trend of fugacity coefficient is slightly higher than the original trend; At 308.15 K and 318.15 K, when the pressure exceeds critical pressure 7.38 MPa, CO_2_ gradually enters the supercritical state, the trend of fugacity coefficient is slightly lower than the original trend. The order of the fugacity coefficient of various gases affected by temperature and pressure is CO_2_ > O_2_ > CO > N_2_.

#### Fugacity of binary-components

**Figure 4:**
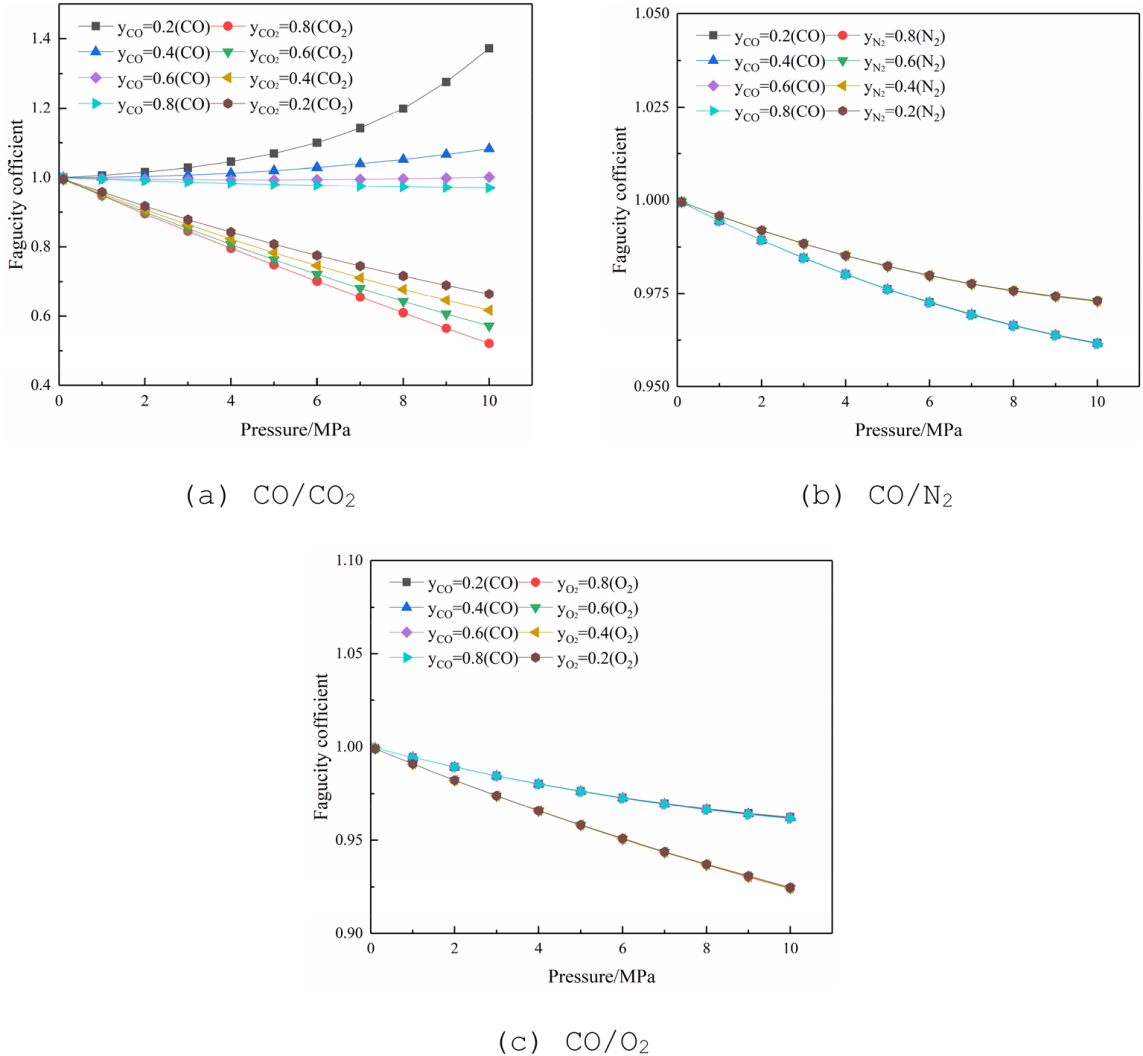
Relationship between fugacity coefficient and pressure of binary-components at 298.15 K.

At 298.15 K, the fugacity and pressure relationship of the binary-components of CO with other gases are shown in Fig. 4. According to the calculation of the binary-components Peng-Robinson formula^32^, it can be found that when CO and CO_2_ are mixed, the fugacity coefficients are greatly affected by the molar ratio. Under a condition of a certain molar ratio, with the increase of pressure, the fugacity coefficient of CO increases, and the fugacity coefficient of CO_2_ decreases; under a condition of the same pressure, with the increase of temperature, the fugacity coefficients of CO and CO_2_ both decrease. This is because the CO_2_ fugacity coefficient is much more affected than CO in the same pressure and temperature. While CO and O_2_ or N_2_ form a mixture, the molar ratio has little effect on the fugacity coefficient, and the fugacity point diagrams of the gas at different ratios coincide.

## Results and discussion

The “Sorption” module in the molecular simulation software was used to simulate the adsorption of four gases on the lignite coal molecular supercell structure. Based on the GCMC (Grand Classic Monte Carlo) method^33^, the adsorption isotherms of single-components and binary-components at different temperature conditions are obtained. The “Sorption” module determines the adsorption position based on the annealing principle, selects the Metropolis algorithm, selects the Dreiding force field^34^ under the charge balance method (QEq)^35^, selects the Ewald & Group method^36^ to calculate the electrostatic force, the Atom-based Method^36^ to calculate van der Waals force. Determine the number of balance steps as 10^6^ and the number of production steps as 2*10^6^ to obtain accurate balance data through the sampling method. When C/D Ratio in the logfile is close to 1, it can be considered that the simulation is accurate.

The unit of adsorption capacity obtained from the simulation results is [average molecules/cell], which is converted to [mmol/g] by Eq. (1):

1 $$ Uptake\;({\text{mmol}}/{\text{g}}) = \frac{Loading\,molecules}{{M_{rcell} ({\text{g}}/{\text{mol}})}} \times 10^{3} $$

where $M_{rcell}$ is the relative molecular mass of the adsorbent in the unit cell.

### Single-component adsorption isotherms of CO, CO_2_, N_2_, and O_2_

**Figure 5:**
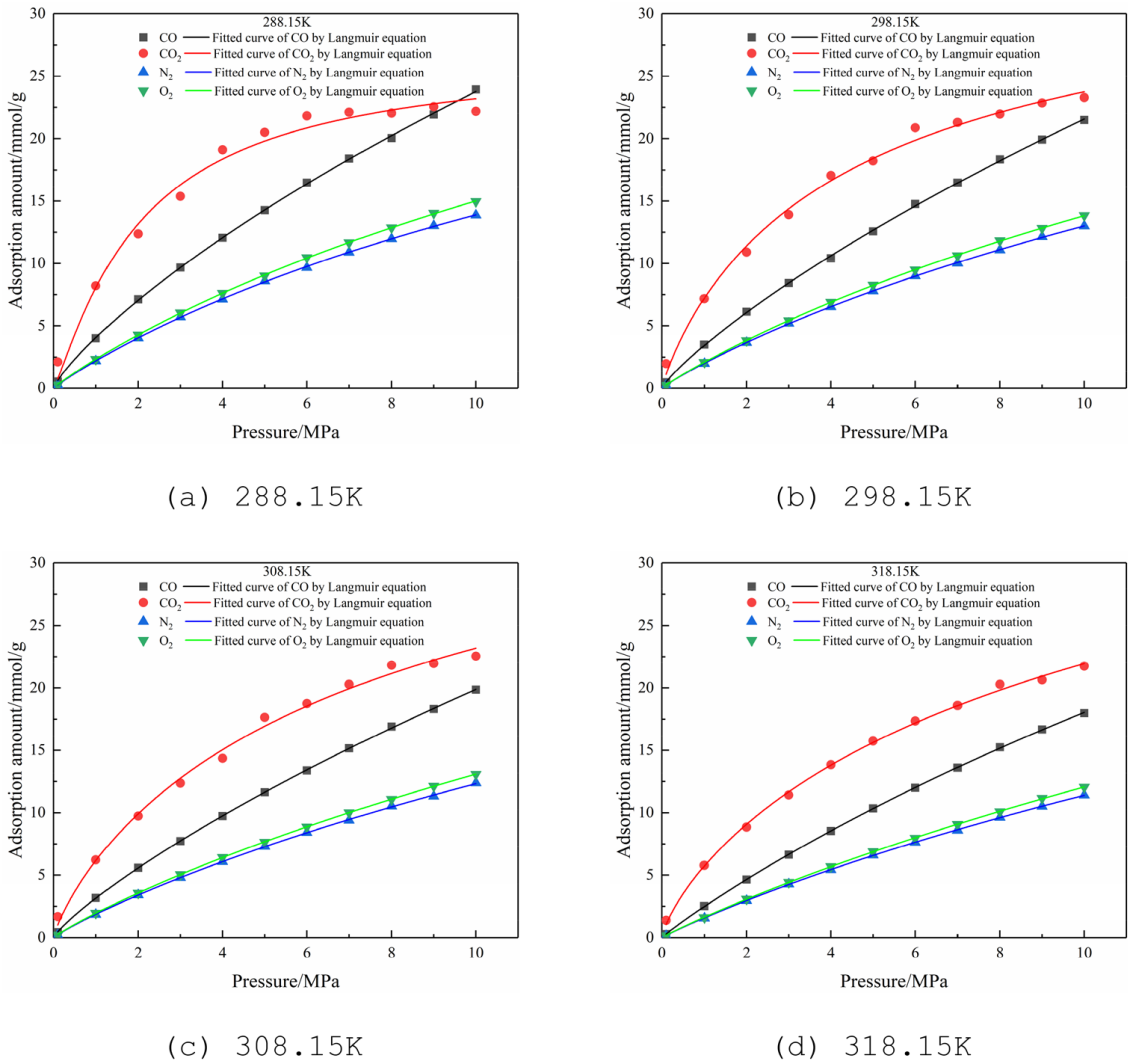
Adsorption isotherms of CO, CO_2_, N_2_, and O_2_ at different temperatures.

It shows the adsorption isotherms of CO, CO_2_, N_2_, and O_2_ at 288.15–318.15 K and 0–10 MPa in Fig. 5. The data is fitted to the adsorption isotherm by the Langmuir equation^37,38^. The Langmuir fitting formula is:

2 $$ y = abx/(1 + bx) $$

where *a* represents the limit adsorption capacity of the adsorbate when the pressure tends to infinity, mmol/g; *b* is the adsorption constant, MPa^-1^. The simulation results are shown in Table 2, where R^2^ is the fitting accuracy.

**Table 2:** Langmuir fitting parameters of single-component adsorption at different temperatures.

Temperature/K	Gas	a	b	R^2^
288.15	CO	61.93359	0.06107	0.99854
	CO_2_	28.96694	0.41977	0.98319
	N_2_	36.32608	0.06158	0.99976
	O_2_	41.26188	0.05677	0.99976
298.15	CO	64.33922	0.04970	0.99886
	CO_2_	32.34059	0.26936	0.99339
	N_2_	36.94775	0.05390	0.99970
	O_2_	40.77887	0.05097	0.99980
308.15	CO	59.87083	0.04898	0.99901
	CO_2_	34.76296	0.19456	0.99268
	N_2_	37.32935	0.04887	0.99952
	O_2_	41.12169	0.04631	0.99969
318.15	CO	67.21716	0.03651	0.99984
	CO_2_	33.85058	0.17696	0.99609
	N_2_	40.78997	0.03860	0.99986
	O_2_	46.83634	0.03454	0.99985

Under the same temperature and pressure conditions, the order of the adsorption capacity of different gases from large to small is CO_2_ > CO > O_2_ > N_2_ (this sequence conforms to the general situation in 0–10 MPa, but at 288.15 K, after 9.5 MPa, the CO adsorption capacity larger than CO_2_), as shown in Fig. 5. The adsorption capacity of different gases is related to their physical properties. CO_2_, O_2_, and N_2_ are non-polar molecules, and the adsorption capacity is positively related to the critical temperature and critical pressure of the three gases, as shown in Table 1, while CO is a polar molecule with a coordination bond in the molecule, so its adsorption capacity is stronger than N_2_ and O_2_, while the critical temperature of CO_2_ is much greater than that of CO, which is dominant. Finally, the order of adsorption capacity of CO_2_ > CO > O_2_ > N_2_ was got.

Besides, comparing the CO adsorption capacity at different temperatures, it can be found that with the temperature increasing, the adsorption capacity is decreasing, which is also adaptable in the adsorption characteristics of other gases. It can be seen that the oxidation of the residual coal in the goaf will not only produce CO but also cause the desorption of the original CO in the coal. Therefore, it is also necessary to take fire-prevention measures in mines whose coal seams have a large amount of original CO.

### Adsorption isotherms of binary-components

#### Adsorption isotherms of binary-component of CO and CO_2_

**Figure 6:**
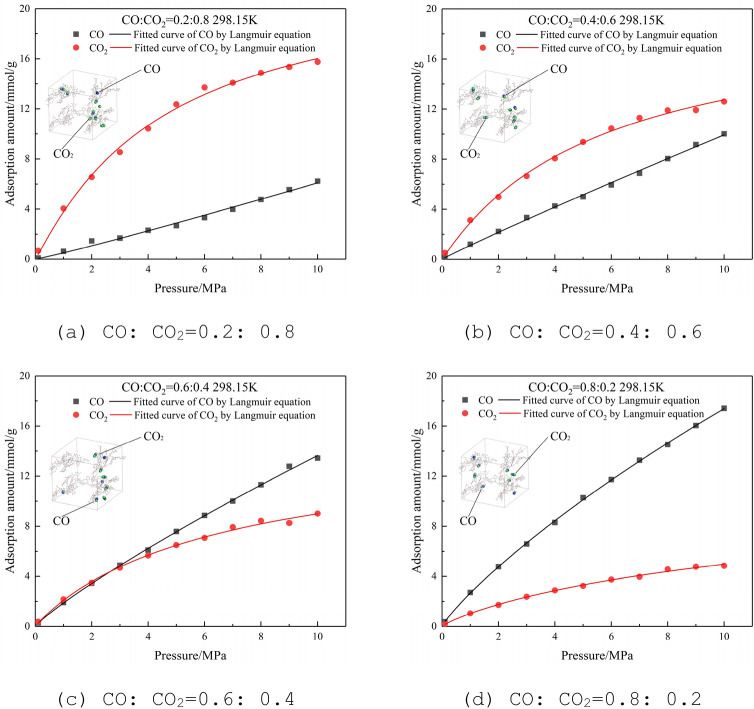
Adsorption isotherms of CO and CO_2_ at different molar ratios at 298.15 K.

At a temperature of 298.15 K and a pressure of 0–10 MPa, CO and CO_2_ are mixed at a molar ratio of 0.2:0.8, 0.4:0.6, 0.6:0.4, and 0.8:0.2, and the Langmuir adsorption isotherm obtained is shown in Fig. 6. It can be seen that the adsorption amount of CO and CO_2_ on the lignite molecular supercell has an obvious relationship with the molar ratio of the binary mixture. The competitive adsorption capacity of the two gases is very close. Comparing molar ratio 0.4 and 0.6 of CO, when the pressure is low, the adsorption capacity of CO_2_ is better than that of CO; comparing molar ratio 0.2 and 0.8 of CO, when the pressure is high, the adsorption capacity of CO_2_ is inferior to CO.

In the actual production of coal mines, it is generally difficult to reach a high-pressure state in goaf by pre-injecting CO_2_ to prevent fire. Therefore, it is believed that the adsorption capacity of CO_2_ is greater than that of CO in actual production. That is, CO_2_ is not suitable as a fire-fighting gas in coal mines whose coal seams have a large amount of original CO. CO_2_ with strong competitive adsorption capacity will occupy the adsorption site, resulting in a large amount of CO desorption in goaf and diffusion in the working surface.

#### Adsorption isotherms of binary-component of CO and N_2_

**Figure 7:**
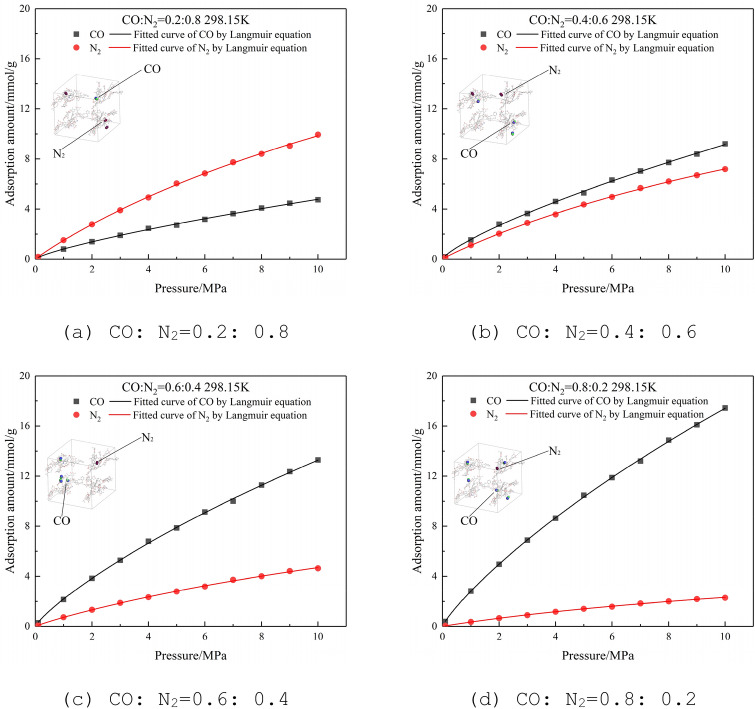
Adsorption isotherms of CO and N_2_ at different molar ratios at 298.15 K.

At a temperature of 298.15 K and a pressure of 0–10 MPa, CO and N_2_ are mixed at a molar ratio of 0.2:0.8, 0.4:0.6, 0.6:0.4, and 0.8:0.2, and the Langmuir adsorption isotherm obtained is shown in Fig. 7. It can be seen that the adsorption amount of CO and N_2_ on the lignite molecular supercell has an obvious relationship with the molar ratio of the binary mixture. The adsorption capacity of CO is better than that of N_2_. When the molar ratio is 0.2:0.8, the adsorption amount of N_2_ is greater than that of CO. When the molar ratio of CO is greater than 0.4, the adsorption amount of CO is significantly higher than that of N_2_.

In the actual production of coal mines with a large amount of original occurrence CO, N_2_ injection is not used as much as possible to prevent fire. Although the adsorption capacity of N_2_ is lower than that of CO, it will still interfere with the adsorption of CO and cause part of the CO to desorb; If the goaf must be pre-injected to prevent fire, the effect of N_2_ is better than CO_2_. The relatively weak competitive adsorption capacity of N_2_ can not only reduce the oxygen concentration in the goaf but also occupy a few adsorption sites.

#### Adsorption isotherms of binary-component of CO and O_2_

**Figure 8:**
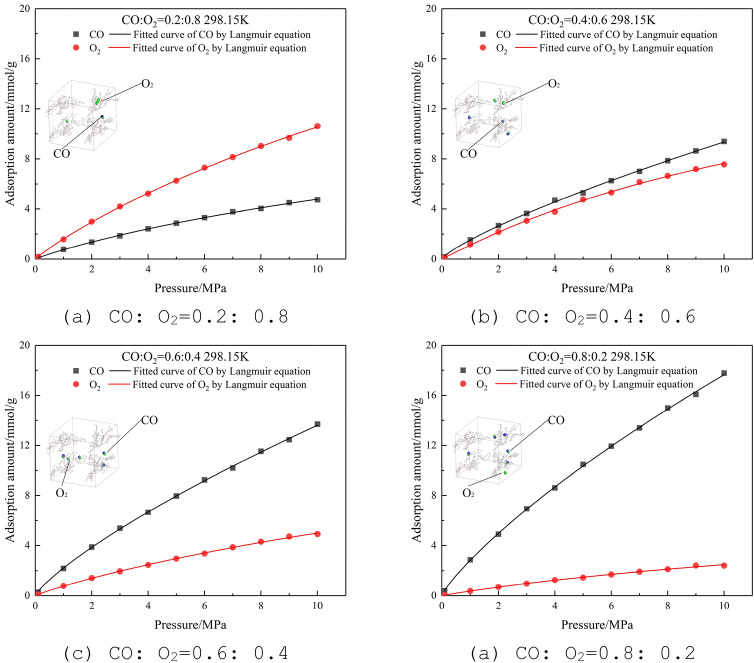
Adsorption isotherms of CO and O_2_ at different molar ratios at 298.15 K.

At a temperature of 298.15 K and a pressure of 0–10 MPa, CO and O_2_ are mixed at a molar ratio of 0.2:0.8, 0.4:0.6, 0.6:0.4, and 0.8:0.2, and the Langmuir adsorption isotherms obtained are shown in Fig. 8. It can be seen that the result is similar to CO and N_2_ on the lignite molecular supercell. The adsorption capacity of CO is better than that of O_2_. When the molar ratio is 0.2:0.8, the adsorption amount of O_2_ is higher than that of CO; When the CO molar ratio is greater than 0.4, the CO adsorption amount is significantly higher than O_2_.

In the actual production of coal mines with a large amount of original CO, the air leakage control at the working faces and other locations should be strengthened to reduce fresh air entering the goafs. Although the adsorption capacity of O_2_ is not as good as that of CO, it will still interfere with the adsorption of CO and cause some CO to desorb.

### Single-component adsorption isosteric heats

**Figure 9:**
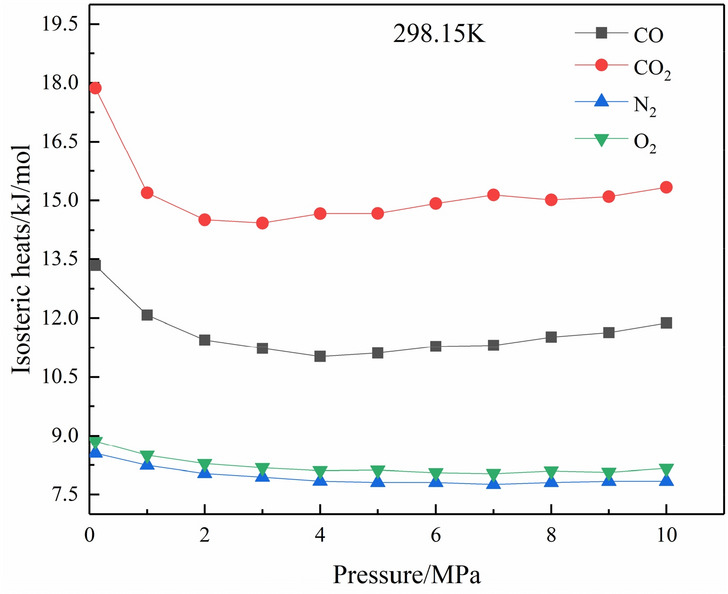
Adsorption isosteric heats of CO, CO_2_, N_2_, and O_2_ at 298.15 K.

**Figure 10:**
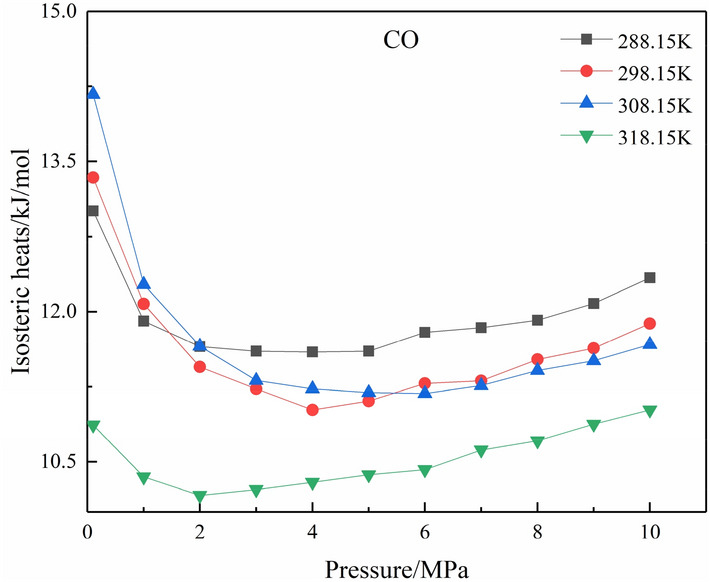
Adsorption isosteric heats of CO at 288.15–318.15 K.

The isosteric heat of adsorption reflects the strength of the interaction between adsorbate and adsorbent. The higher the isosteric heat of adsorption, the stronger the interaction between adsorbate and adsorbent. When the isosteric heat of adsorption is less than 41.84 kJ/mol, the adsorption form is only physical adsorption, no chemical adsorption. When the temperature is 298.15 K and the pressure is 0–10 MPa, the isosteric heats of adsorption of CO, CO_2_, N_2_, and O_2_ are shown in Fig. 9. As the pressure increases, the adsorption heat of each gas first decreases and then increases, but the maximum value is still at the lowest pressure (0.1 MPa) and less than 41.84 kJ/mol, indicating that each gas is adsorbed at the initial pressure and has the strongest interaction with coal, and there is no chemical adsorption. The isosteric heat of CO adsorption at temperatures of 288.15, 298.15, 308.15, and 318.15 K and pressure 0–10 MPa is shown in Fig. 10. It can be seen that in the pressure 0–2 MPa and the temperature 308.15 K, the isosteric heat of adsorption is the largest, and CO has the strongest interaction with coal; in the pressure 2–10 MPa, when the temperature is 288.15 K, the isosteric heat of adsorption is the largest, and CO has the strongest interaction with coal; when the temperature is 318.15 K, the isosteric heat of adsorption is always smallest, which further shows that high temperature is not conducive to CO adsorption, that is, it will cause CO desorption.

## Conclusion

1. Lignite molecules have different structures at different temperatures. In this paper, the lignite molecules are annealed and optimized at the normal temperature range (288.15, 298.15, 308.15, and 318.15 K) of working surfaces, and the lignite unit cell structure under the temperature step is obtained. Through the adsorption study of CO, CO_2_, N_2_, and O_2_, the Langmuir equation of adsorption capacity and pressure load was found. The order of adsorption capacity of different gases at 0–10 MPa pressure is CO_2_ > CO > O_2_ > N_2_.
2. For the CO/CO_2_ binary-components, the competitive adsorption capacity of CO_2_ is stronger than that of CO; for the CO/N_2_ and CO/O_2_ binary-components, even if the molar ratio of N_2_ or O_2_ reaches 0.6, its adsorption capacity is still significantly smaller than that of CO, indicating that the competitive adsorption of CO in the binary-component is stronger than that of N_2_ or O_2_.
3. For coal seams with a large amount of original CO, coal mines should adopt other measures to prevent fires in goafs, such as grouting. It is best not to use N_2_ or CO_2_ injection to prevent fires. At the same time, air leakage control in goafs should be strengthened. To prevent CO_2_, N_2_, and O_2_ from occupying adsorption sites and prevent the oxidation and heating of the residual coal, so that prevent the adsorbed CO in residual coal from desorbing and diffusing to the work face.

## Supplementary Information

Supplementary Information 1.

## Data Availability

All data generated or analysed during this study are included in this published article (and its Supplementary Information files).
